# Patterns and trends of postpartum family planning in Ethiopia, Malawi, and Nigeria: evidence of missed opportunities for integration

**DOI:** 10.3402/gha.v8.29738

**Published:** 2015-11-09

**Authors:** Sennen Hounton, William Winfrey, Aluisio J. D. Barros, Ian Askew

**Affiliations:** 1 United Nations Population Fund, New York, NY, USA; 2 Avenir Health, Glastonbury, CT, USA; 3 International Center for Equity in Health, Federal University of Pelotas, Capão do Leão, Brazil; 4 Population Council, Nairobi, Kenya

**Keywords:** postpartum contraception, integration, maternal and newborn health services, sub-Saharan Africa

## Abstract

**Background:**

The first 12 months following childbirth are a period when a subsequent pregnancy holds the greatest risk for mother and baby, but also when there are numerous contacts with the healthcare system for postnatal care for mother and baby (immunisation, nutrition, etc.). The benefits and importance of postpartum family planning are well documented. They include a reduction in risk of miscarriage, as well as mitigation of (or protection against) low birth weight, neonatal and maternal death, preterm birth, and anaemia.

**Objectives:**

The objectives of this paper are to assess patterns and trends in the use of postpartum family planning at the country level, to determine whether postpartum family planning is associated with birth interval and parity, and to identify the health services most closely associated with postpartum family planning after adjusting for socio-economic characteristics.

**Design:**

Data were used from Demographic and Health Surveys that contain a reproductive calendar, carried out within the last 10 years, from Ethiopia, Malawi, and Nigeria. All women for whom the calendar was completed and who gave birth between 57 and 60 months prior to data collection were included in the analysis. For each of the births, we merged the reproductive calendar with the birth record into a survey for each country reflecting the previous 60 months. The definition of the postpartum period in this paper is based on a period of 3 months postpartum. We used this definition to assess early adoption of postpartum family planning. We assessed variations in postpartum family planning according to demographic and socio-economic variables, as well as its association with various contact opportunities with the health system [antenatal care (ANC), childbirth in facilities, immunisation, etc.]. We did simple descriptive analysis with tabular, graphic, and ‘equiplot’ displays and a logistic regression controlling for important background characteristics.

**Results:**

Overall, variation in postpartum use of modern contraception was not affected over the years by age or marital status. One contrast to this is in Ethiopia, where the data show a significant increase in uptake of postpartum contraception among adolescents from 2005 to 2011. There are systematic and pervasive equity issues in the use of modern postpartum family planning by education level, place of residence, and wealth quintile, especially in Ethiopia where the gaps are very large. Disaggregation of data also point to significant sub-national variations. After adjusting for socio-economic variables, the most consistent health sector services associated with modern postpartum contraception are institutional childbirth and child immunisation. ANC is less likely to be associated with the use of modern postpartum family planning.

**Conclusion:**

Postpartum use of modern family planning has remained very low over the years, including for childbearing adolescents. Our results indicate that improving postpartum family planning requires policies and strategies to address the inequalities caused by socio-economic factors and the integration of family planning with maternal and newborn health services, particularly with childbirth in facilities and child immunisation. Scaling up systematic screening, training of providers, and generation of demand are some possible ways forward.

**Paper context**

Postpartum contraception remains very low in sub-Saharan Africa. This paper investigates missed opportunities in the use of postpartum family planning in association with the health services, after adjusting for socio-economic characteristics. There are systematic and pervasive equity issues in the use of modern postpartum family planning. After adjusting for socio-economic variables, the most consistent health sector services associated with modern postpartum contraception are institutional childbirth and child immunisation. The results point to missed opportunities for better integration of maternal, newborn, and family planning services.

Achieving universal access to voluntary family planning and contraception services requires enabling policy and political environments, quality services, demand generation interventions, and integration of health systems to address missed opportunities. A key marker for high quality and integrated services is postpartum family planning (1). Postpartum family planning refers to the prevention of unintended and/or closely spaced pregnancies in the period after delivery. The first 12 months following childbirth is a period when a subsequent pregnancy holds the greatest risk for mother and baby (1), but also when there are numerous health service contacts for postnatal care for both mother and baby (for instance, child immunisation, nutrition, etc.).

The benefits and importance of postpartum family planning are well documented and include, at the individual level, reduction in the risk of miscarriage, low birth weight, neonatal death, maternal death, preterm birth, anaemia, and premature rupture of membranes (2, 3). The risks of early neonatal, neonatal, and infant deaths are high for subsequent births between the first 9 and 18 months after the end of a pregnancy (3, 4). Moreover, family planning could avert more than 30% of maternal deaths and 10% of child deaths if pregnancies were spaced more than 2 years apart (5).

It is thus critical that the health system does not miss any opportunities to offer women postpartum family planning information and services to ensure healthy outcomes for mothers and babies and to empower women to choose when to have subsequent children as well as how many. At the global level, thousands of under-five child deaths could be prevented by ensuring appropriate birth spacing (5). In recent reviews on the use of family planning in the postpartum period, antenatal care (ANC) visits and skilled birth attendance were found to be associated with postpartum family planning (6, 7). Similarly, wealth, education, and place of residence were associated with family planning in the postpartum period. These correlations may be associated with the modality of delivery of family planning services (through health facilities or household-based by community health workers) or with the populations in terms of who they are (wealthiest vs. poorest) rather than the covariates themselves. Moreover, one can expect that high-parity births would be more likely to be followed by contraceptive use. This paper assesses between- and within-country variations for three countries – Ethiopia, Malawi, and Nigeria – to determine whether postpartum family planning is associated with birth intervals and parity and to identify the health services most closely associated with postpartum family planning (adjusting for socio-economic characteristics).

## Data and methods

The data used in this study are from the most recent Demographic and Health Surveys (DHS) in Ethiopia (2005 and 2011), Malawi (2004 and 2010), and Nigeria (2008 and 2013). The fieldwork for the surveys took place between 2004 and 2013 and they contain a reproductive calendar. These countries were chosen because they have a low or moderate level of family planning use and have conducted two recent surveys with the complete reproductive health calendar, which is necessary for analysing postpartum family planning for a large sample of births. They also capture the diversity of sub-Saharan African countries in terms of geography, culture, religious groups, level of decentralisation, population size, contraceptive prevalence, languages, regions, and economic indicators. All women aged 15–49 years for whom the calendar was completed and who had given birth in the previous 60 months were included in the analysis. The complete reproductive calendar typically records, for each of the 60 months preceding the interview, all pregnancies, births, and terminations, as well as use of family planning (7). We merged the DHS women's data set (individual recode, which includes the reproductive calendar) with the DHS birth record for each of the births recorded in a survey for the last 60 months for each country.

The definition of the postpartum period in this paper is based on a period of 3 months postpartum. We used this definition to assess early adoption of postpartum family planning. In fact, the reproductive calendar is based upon the women's memory of the past 60 months. Initiation of family planning might be remembered relatively accurately if family planning was initiated immediately postpartum. Rather than needing to remember a particular month, a woman could remember that she started using family planning immediately following birth, an event easily recalled or likely to be documented. A longer postpartum period is likely to make the users of postpartum family planning similar to all users of family planning, whereas the focus in this study is on early adoption (7).

Modern family planning methods as defined in the DHS include female sterilisation, male sterilisation, pills, intrauterine devices, injectables, implants, male condoms, female condoms, diaphragms, foam/jelly, the lactational amenorrhoea method, and emergency contraception. Initiation of modern family planning (defined as involving the use of modern methods of contraception) during the first 3 months after delivery was disaggregated by demographic and socio-economic factors, parity, and birth intervals, as well as opportunistic use of the health system (ANC, institutional delivery, and child immunisation). The average prevalence in the latest DHS for ANC, skilled attendance at delivery, and child immunisation (third dose of diphtheria–tetanus–pertussis, DTP3) are: 43, 10, and 37% for Ethiopia; 95, 71, and 93% for Malawi; and 61, 38, and 38% for Nigeria, respectively.

Descriptive results are presented in tabular form, as maps, or via equiplots to best illustrate trends or disparities. Logistic regressions were also conducted for each survey analysed. This analysis explored the factors of service delivery use that were most correlated with postpartum family planning, adjusting for socio-economic factors. All of the analyses were performed with Stata 13.0 statistical software, taking into account the design characteristics of the surveys. Ethics procedures were the responsibility of the institutions that commissioned, funded, or administered the surveys.

## Results

**Fig. 1:**
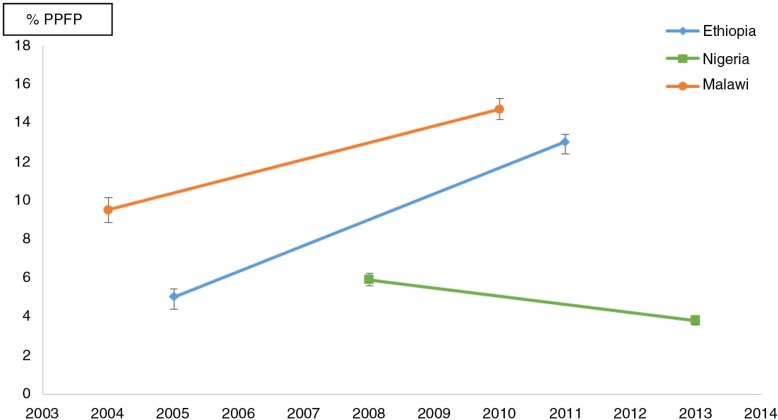
Trends in average percent modern postpartum family planning use (% PPFP) at 3 months, all women 15–49 years old, with 95% confidence intervals in Ethiopia, Malawi, and Nigeria. Source: DHS.

As can be seen in Fig. 1, postpartum family planning has improved in Ethiopia and Malawi over the last two surveys, with a 160 and 55% increase in the national average from the 5 and 9.5% baseline, respectively. In contrast, in Nigeria, not only there was no increase from 2008 to 2013, but postpartum family planning prevalence has decreased from an already low level of 5.9–3.8%.

### Disaggregation of modern postpartum family planning by age and marital status

**Fig. 2:**
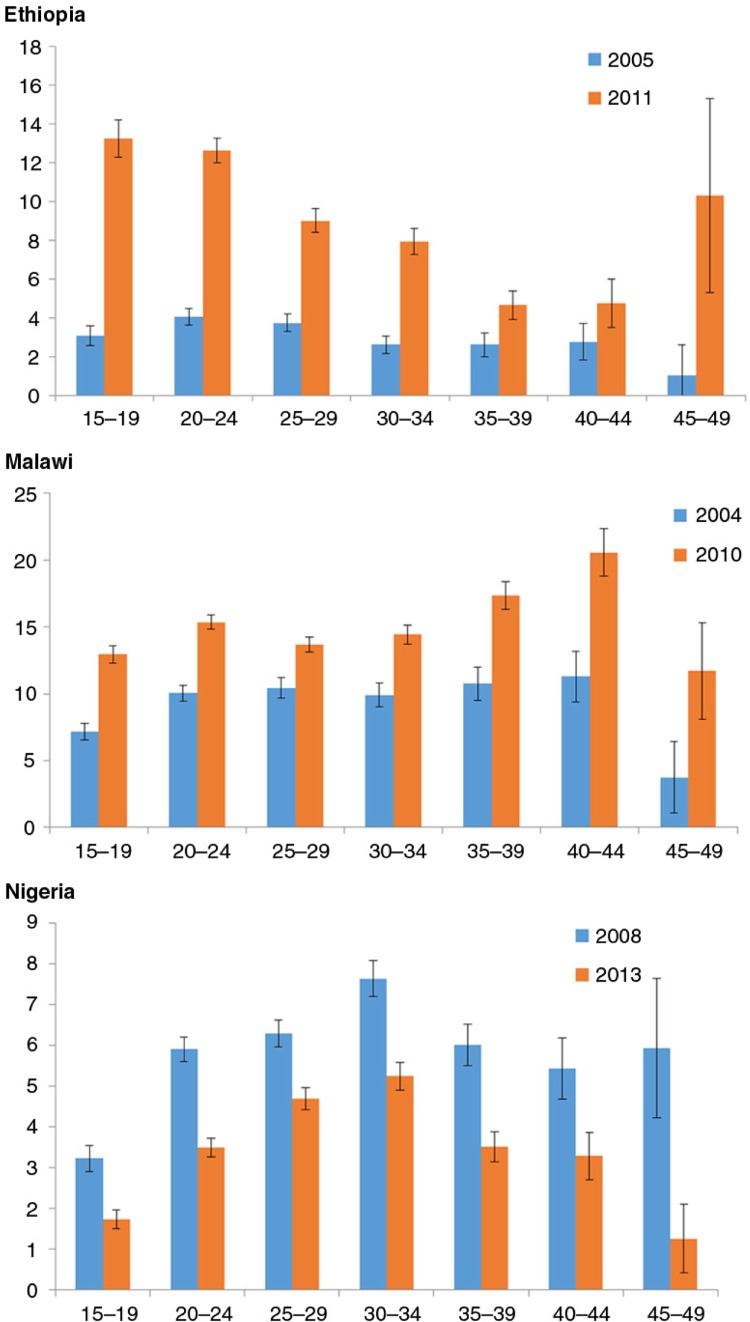
Distribution of modern postpartum family planning at 3 months, all women 15–49 years old, by age, with 95% confidence intervals in Ethiopia, Malawi, and Nigeria. Source: DHS.

Figure 2 presents the differentials by 5-year age groups in the three countries. With the exception of the 40–44 age group in Ethiopia, there were statistically significant differences across all age groups over time in both Ethiopia and Malawi. In Ethiopia, the highest increases in 2011 were observed among adolescents (15–19), youth (20–24), and women aged 45–49, while in Malawi, the highest increases were observed for women above 35 years old.

Among adolescents 15–19 years old, modern postpartum contraception has increased by 4.3 and 1.8 times in Ethiopia and Malawi, respectively. In Nigeria, there has been a systematic decrease in contraceptive use by all age groups, and there was no significant difference in the decrease across the age groups.

Regarding marital status, there was a skewed distribution towards marital status in the analysis samples, with 98.5, 96.9, and 97.3% of women in a union in Ethiopia, Malawi, and Nigeria, respectively. Births outside of unions are thus not common in these three countries. Using the latest DHS, we did not find any significant variation in postpartum family planning use by marital status in the three countries (data not presented).

### Distribution of modern postpartum family planning by socio-economic status

We explored variations in postpartum use of modern contraception by education level, place of residence (urban vs. rural), and wealth quintile. The data are presented in Table 1 and equiplots are constructed for the three countries (Fig. 2). Overall, there is a strong and statistically significant association between the use of modern postpartum family planning and education level, place of residence, and wealth quintile across all three countries. The inequalities have increased over the years in Ethiopia and Malawi between women with secondary-level education and above and women with primary-level education or lower. There was no statistically significant effect of education level in Nigeria, where the reduction appeared similar across all levels of education. The patterns of differentials by place of residence and education level were similar to one another, with increased inequalities over time between the urban and rural areas in Ethiopia and Malawi. In Nigeria, the absolute inequality in access and use of modern contraception in the postpartum period remained constant. Finally, regarding differentials by wealth quintile, the use of modern postpartum family planning was significantly higher among the richest quintiles compared to the poorest across all three countries. In Ethiopia, the inequality in the use of modern postpartum family planning increased from 2005 to 2011, from a 12.4% differential to a 28.9% differential between the lowest and highest quintiles. In Malawi and Nigeria, there did not appear to be significant differentials by wealth quintile across the last two surveys.

**Table 1:** Postpartum use of modern family planning at 3 months among women 15–49 years old disaggregated by education, place, and wealth quintile in Ethiopia, Malawi, and Nigeria

	Ethiopia				Malawi				Nigeria			
			
	2005	2005 SDV	2011	2011 SDV	2004	2004 SDV	2010	2010 SDV	2008	2008 SDV	2013	2013 SDV
Education												
No education	1.6	0.2	6.8	0.3	7.2	0.6	12.7	0.6	2.5	0.2	0.9	0.1
Primary	5.7	0.7	13.9	0.7	10.0	0.4	14.2	0.3	6.7	0.4	4.4	0.3
Secondary >	26.4	1.9	42.3	2.4	13.0	1.2	19.8	0.9	10.7	0.4	8.2	0.3
Place												
Urban	21.2	1.3	34.4	1.2	12.1	1.1	22.1	1.1	10.1	0.4	7.0	0.3
Rural	1.9	0.2	6.3	0.3	9.1	0.3	13.5	0.3	4.1	0.2	2.1	0.1
Wealth quintiles												
Lowest	0.8	0.2	2.7	0.3	6.8	0.6	11.7	0.5	2.8	0.2	0.6	0.1
Second	0.9	0.3	6.6	0.6	7.6	0.6	13.0	0.6	3.4	0.3	1.4	0.2
Middle	2.2	0.4	6.1	0.6	8.8	0.6	14.6	0.6	4.2	0.3	2.5	0.2
Fourth	2.6	0.5	9.5	0.7	10.7	0.8	16.8	0.7	7.0	0.4	6.4	0.4
Highest	13.2	0.9	31.6	1.1	14.9	1.0	18.9	0.9	14.3	0.7	10.5	0.5
All	5.0	0.2	11	0.3	9.5	0.3	14.7	0.3	5.9	0.1	3.8	0.2

SDV, standard deviation of the mean percent using modern family planning at 3 months postpartum in the latest Demographic and Health Surveys; primary, primary completed; secondary >, above primary school.

### Distribution of modern postpartum family planning by parity and birth interval

Table 2 presents the pattern of modern postpartum contraception use in the three countries by parity and birth interval. The use of modern postpartum contraception remains low in Nigeria regardless of levels of parity. There was no significant difference attributed to parity in Malawi. In Ethiopia, the use of modern postpartum family planning varied significantly by parity and was higher for low parity with a drop-off by half in the use of modern postpartum family planning for parity of four and above. For birth interval, apart from a slight increase in modern postpartum contraception use among women with short birth intervals in Malawi, there was no significant effect of birth interval.

**Table 2:** Prevalence of modern postpartum family planning at 3 months among women 15–49 years old by parity and birth interval (preceding birth)

	Ethiopia 2011 DHS		Malawi 2010 DHS		Nigeria 2013 DHS	
			
Countries Covariates	Mean postpartum family planning	SDV	Mean postpartum family planning	SDV	Mean postpartum family planning	SDV
Parity						
One	16.8	0.9	13.1	0.6	3.7	0.3
Two or three	11.8	0.6	14.7	0.5	4.7	0.2
Four or five	6.0	0.5	15.2	0.6	3.9	0.3
Six or more	6.1	0.5	15.8	0.7	2.7	0.2
Birth intervals (preceding birth)						
<24 months	8.1	0.7	18.7	0.9	3.2	0.3
≥24 months	8.3	0.4	14.5	0.3	4.1	0.2

DHS, Demographic and Health Survey; SDV, standard deviation of the mean percent using modern family planning at 3 months postpartum in the two latest DHS.

### Sub-national variations in postpartum family planning in Ethiopia (2011), Malawi (2010), and Nigeria (2013)

**Fig. 3:**
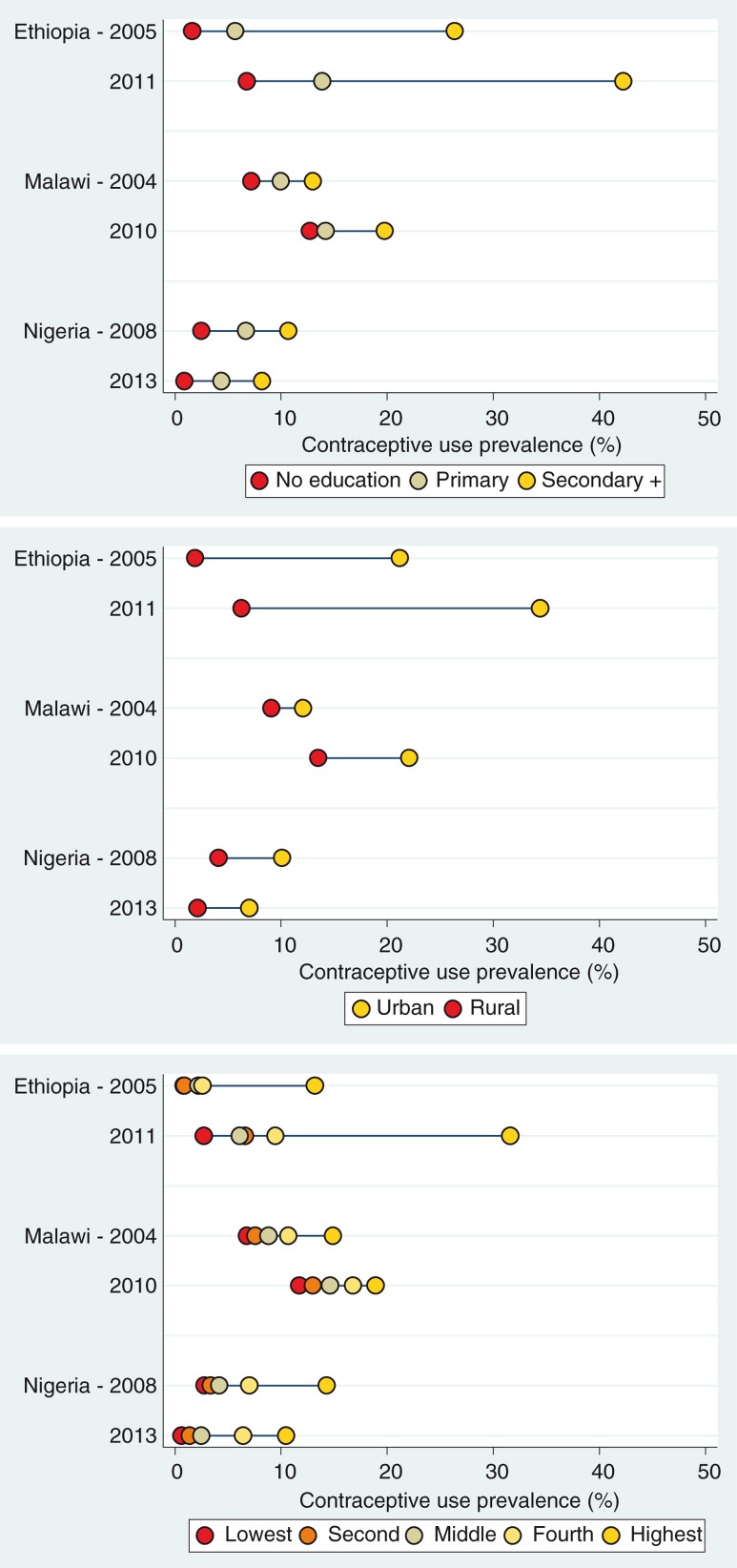
Equiplots of modern postpartum family planning at 3 months, all women 15–49 years old, by education, place, and wealth quintile in Ethiopia, Malawi, and Nigeria.

**Fig. 4:**
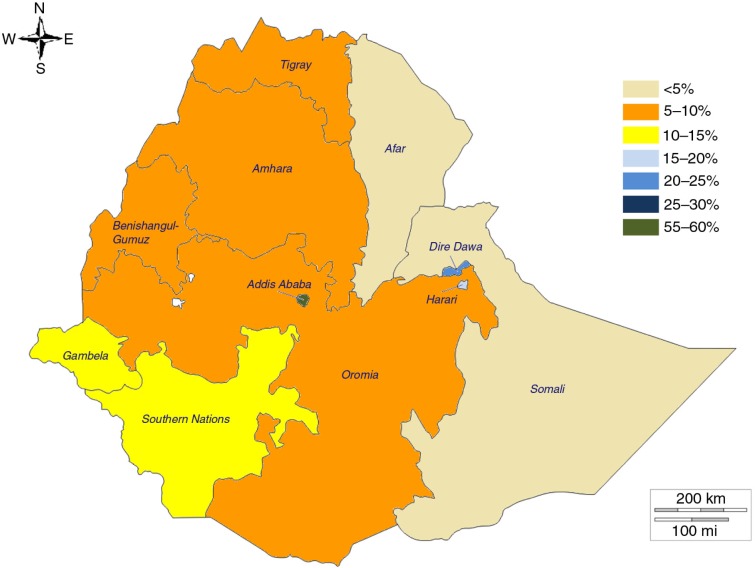
Mapping of prevalence of modern postpartum family planning use at 3 months, all women 15–49 years old, by region, Ethiopia. Source: 2011 DHS.

**Fig. 5:**
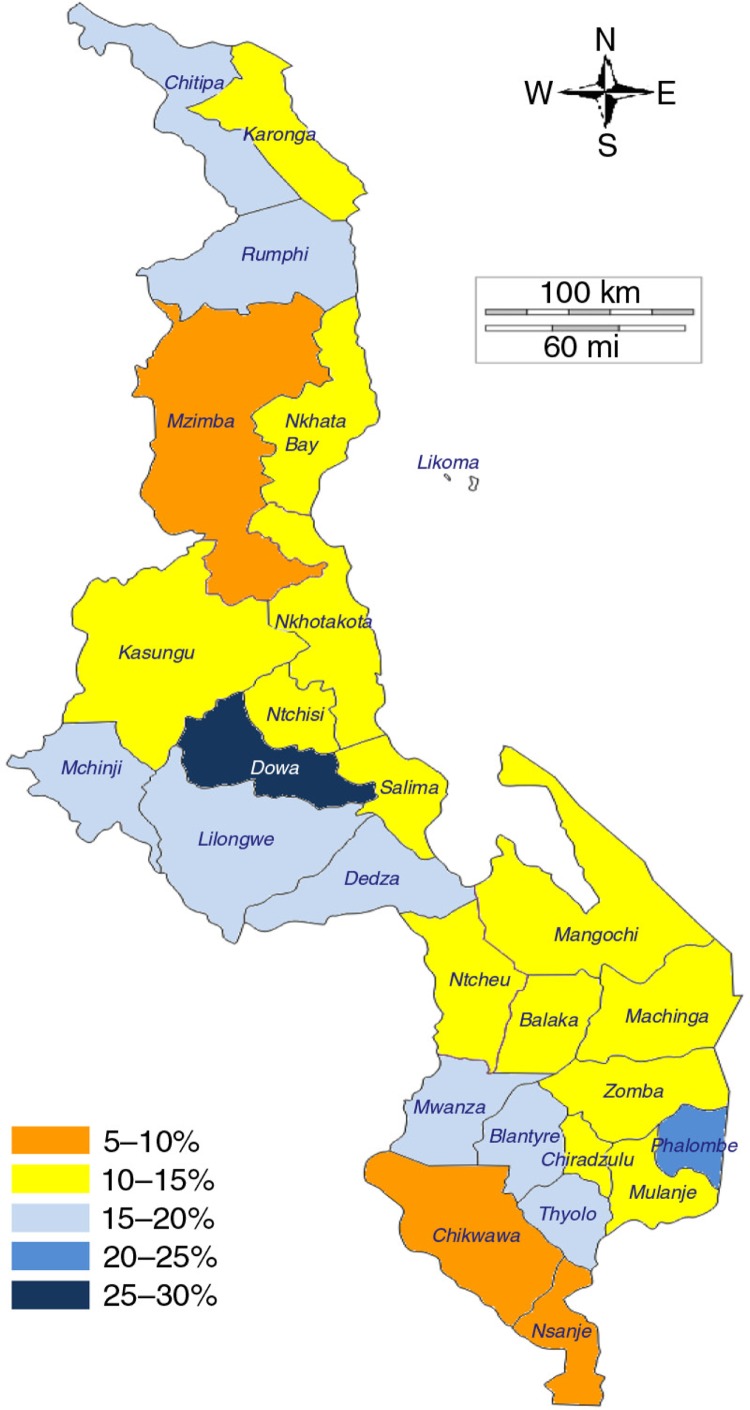
Mapping of prevalence of modern postpartum family planning use at 3 months, all women 15–49 years old, by district, Malawi. Source: 2010 DHS.

There were very large regional differences in the proportions of modern postpartum contraception use in the three countries (Figs. 3–5), although the low prevalence of modern postpartum family planning across the three countries mirrors a generally low level of modern contraception in sub-Saharan Africa (8). Our study revealed that in Ethiopia 55% of childbearing women adopted modern postpartum family planning in the Addis Ababa region, followed by the Dire Dawa region (a city administration similar to Addis Ababa), where 21% of childbearing women adopted modern postpartum family planning in the 3 months following childbirth.

In Malawi, there were between and within variations across the three regions of the country. In the Central, Southern, and Northern regions, 17, 13, and 11% of childbearing women had used modern postpartum family planning, respectively. However, when we disaggregated the data by region, we saw a significant difference between the Dowa district and the Nkhotakota district in the Central region, with prevalence at 29 and 12%, respectively. The same could be observed between the Rumphi district and the Mzimba district in the Northern region, with prevalence at 17 and 8%, respectively. A similar pattern was observed in the Southern region, pointing to the importance of sub-national analysis.

**Fig. 6:**
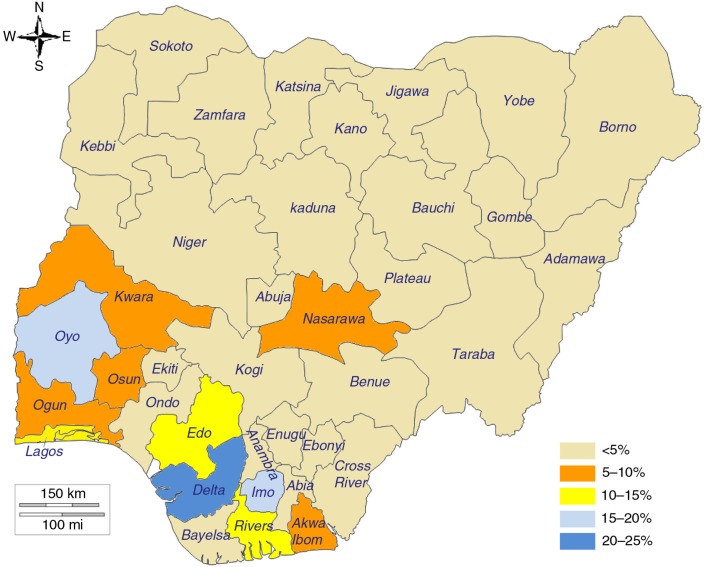
Mapping of prevalence of modern postpartum family planning use at 3 months, all women 15–49 years old, by state, Nigeria. Source: 2013 DHS.

In Nigeria (Fig. 6), the prevalence of modern postpartum family planning was extremely low, with some outlier regions (South South and South West) and states (Delta, Edo, Imo, Lagos, and Oyo) where the prevalence was significantly above the national average. It is interesting to note that these five states were not necessarily the top five states for overall modern contraception use in Nigeria. An inquiry with development partners in Nigeria revealed that the observed differences may be associated with the Nigeria Urban Reproductive Health Initiative programme, which targeted urban slum dwellers in five selected states including Oyo (Ibadan) and Edo from 2009 to 2014 (9). The project utilised a holistic approach for family planning programming in the cities to target communities with a high population of urban poor. Similarly, the Delta and Edo states have benefited from the ACCESS Family Planning Program, which focused on postpartum family planning from 2008 to 2014 using an innovative systematic screening strategy (10, 11). In Malawi, specific investments with a focus on postpartum family planning are fairly recent and differences across the districts may more reflect differences in the socio-economic or demographic factors of the women. In summary, in all three countries, the observed variations point more to variance in opportunities in access and supply or social and cultural behavioural norms.

### Effects of selected health services on adoption of postpartum family planning

Along the continuum of points of contact for postpartum family planning we also explored the association of ANC visits, institutional delivery, and child immunisation (DTP3 as proxy) with postpartum modern contraceptive use. We modelled the variables individually and in a multivariate analysis, adjusting for socio-economic factors. The results are presented in Table 3. After adjusting for covariates, there were statistically significant associations between institutional delivery and child immunisation and the use of modern postpartum family planning across all three countries.

**Table 3:** Multivariate analysis of effect of contacts with the health system on modern postpartum contraception use

Variables	Adjusted OR	Lower CI	Higher CI
Ethiopia (2011)			
Antenatal care (1–3 visits)	1.12	0.89	1.41
Antenatal care (4 visits)^a^	1.92	1.54	2.39
Institutional delivery (yes)^a^	2.04	1.63	2.56
Child immunisation^a^ (completion of DTP3)	1.85	1.56	2.20
Education (primary)^a^	1.63	1.36	1.96
Education (higher)^a^	1.34	1.03	1.75
Residence (urban)^a^	0.68	0.52	0.90
Wealth quintiles – second^a^	3.04	2.02	4.57
Wealth quintiles – third^a^	3.55	2.38	5.30
Wealth quintiles – fourth^a^	4.48	3.06	6.57
Wealth quintiles – fifth^a^	7.48	4.94	11.33
Malawi (2010)			
Antenatal care (1–3 visits)	0.96	0.75	1.23
Antenatal care (4 visits)	1.08	0.85	1.39
Institutional delivery (yes)^a^	1.31	1.16	1.47
Child immunisation^a^			
(completion of DTP3)	1.47	1.29	1.69
Education (primary)	1.05	0.92	1.20
Education (higher)	1.12	0.94	1.34
Residence (urban)^a^	0.66	0.57	0.77
Wealth quintiles – second^a^	1.16	1.01	1.34
Wealth quintiles – third	1.13	0.98	1.30
Wealth quintiles – fourth	1.14	0.98	1.33
Wealth quintiles – fifth^a^	1.20	1.01	1.43
Nigeria (2013)			
Antenatal care (1–3 visits)	0.85	0.63	1.15
Antenatal care (4 visits)	1.15	0.94	1.42
Institutional delivery (yes)^a^	1.24	1.04	1.48
Child immunisation^a^			
(completion of DTP3)	1.34	1.14	1.57
Education (primary)^a^	1.96	1.51	2.53
Education (higher)^a^	2.20	1.70	2.83
Residence (urban)	1.00	0.84	1.18
Wealth quintiles – second^a^	2.83	1.76	4.57
Wealth quintiles – third^a^	3.70	2.30	5.96
Wealth quintiles – fourth^a^	5.81	3.60	9.38
Wealth quintiles – fifth^a^	8.52	5.19	13.99

a Significant at the 5% level. OR, odds ratio; CI, confidence interval; DTP3, diphtheria–tetanus–pertussis. Adjusted OR calculated from a multivariate logistic regression adjusting for socio-economic variables. Reference categories are no antenatal care visit (antenatal care), no education (education), no health facility delivery (skilled attendance), rural area (residence), no completion of DTP3 (child immunisation), and lowest quintiles (wealth quintiles).

The odds of using modern postpartum family planning after delivering at a health facility were, on average, twice as high compared to women who did not deliver at a health facility in Ethiopia, 1.3 times higher in Malawi, and 1.2 times higher in Nigeria. In Ethiopia, the odds of using modern postpartum family planning when a woman completed DTP3 for her child were twice that of women who did not. The strength of the associations were more modest, but still statistically significant, in Malawi and Nigeria. In general, we did not find that ANC was consistently associated with the adoption of postpartum modern contraception after adjusting for socio-economic variables.

## Discussion

### Demographic and socio-economic factors

The rationale and goal of family planning is to empower women and childbearing adolescents to decide on contraceptive methods, adopt the method of choice, and use this method for 2 years or longer, depending on the reproductive intentions of the woman or couple. Although the level of use of modern postpartum family planning was low across the three countries, the positive change (particularly among adolescents) in Ethiopia and Malawi is encouraging. The level of contraception use in the immediate postpartum period was lower than the overall modern contraception use among sexually active and childbearing adolescents (2, 12). This constitutes an increase in births, which may lead to increased unwanted pregnancies, high fertility, and subsequent high risks for maternal mortality, fistula (leading to continuous urinary or faecal incontinence), and poverty. We did not observe any effect of marital status on the postpartum use of modern contraception. The socio-economic characteristics (education, place of residence, and wealth quintile) of the women were significantly associated with the use of modern contraception in the postpartum period. However, the overall results of our analysis imply that other determinants beyond socio-economic status may be at play.

### Effects of parity and birth spacing

Apart from Ethiopia, where we observed a variation by parity, it was surprising to find no significant association between parity or birth interval and modern contraception use in the postpartum period. The expectation among some that high parity births would be more likely followed by contraceptive use is not borne out in any of the three settings. It is possible that high parity is an indication that a woman is, in fact, not likely to use family planning as she has not controlled her fertility before and therefore will continue that pattern. It is also possible that couples who have unplanned pregnancies continue to have unplanned pregnancies throughout the reproductive health lifecycle or that, once married, women are under pressure to have many children. Alternatively, maybe parity and birth spacing are not among the best predictors or determinants of modern postpartum contraception use in family planning. Regardless, given the low postpartum contraceptive use, there is increased vulnerability to unplanned pregnancy for individuals and couples, and particularly adolescent girls, in the three countries after childbirth.

### Sub-national variations

We observed sub-national variations at the state, regional, and district levels across all three countries. These variations highlight the importance of disaggregated data for evidence-based policy making and programme design. The difference between rural and urban areas could partly account for some of these differences, but the results also point to other plausible explanatory factors, as several districts or states within a region have a very different prevalence of use of modern contraception in the postpartum period. These other factors may reflect differences in supply (performance of the health system) and demand (including culture and social norms within communities).

### Effect of contact points with the health system

We did not directly analyse the effect of variance in the supply of contraceptives in this paper, although the difference between urban and rural areas is a proxy for supply and access, as supply and access are generally better in urban areas and for the wealthiest women. The coverage of opportunistic contacts with the health system (ANC, skilled birth at delivery, child immunisation) varies across the three countries. When exploring the association of opportunistic contacts with the health system, we did not see a consistent relationship between ANC visits (irrespective of the number of the visits) and use of modern contraception in the postpartum period across countries. Given the association between ANC and institutional delivery (13–15), the result may reflect the absence of provision of family planning information and services during ANC. It may also indicate that a woman does not act on information unless it is given to her at a moment when she can act upon it effectively. In addition, proper ANC visits, when well conducted, could be associated with effective postnatal care. The lack of capacity and integration of maternal health services with family planning services results in missed opportunities. In this analysis, we did not assess to what extent community health workers as part of the health system may influence postpartum family planning use. This is partly because we are looking at events that occurred many months or years before such questions were asked. We also explored the effect of either a visit by a family planning health worker or a visit to a health facility by the woman in the previous 12 months and whether information and counsel on family planning were provided to the woman during a visit to a health facility; we did not find an association in another study in Burkina Faso, Ethiopia, and Nigeria (12). The fact that these variables were not found in our analysis to be key determinants for adoption of modern contraception use warrants more investigation via research or surveys that specifically address postpartum family planning.

Overall, the most consistent associations found were institutional delivery and child immunisation. This may reflect the fact that women who use such maternal and newborn health services are also more likely to use postpartum family planning services, or that by attending these services they have more opportunities to be convinced to use them and are provided with a method, or both. The use of DTP3 represents a ‘dose–response’ relationship in the postpartum period, as it corresponds to the third visit after post-delivery discharge and provides plenty of opportunity to inform, educate, and offer postpartum family planning. Giving birth in a facility and child immunisation appear to be critical factors and potentially missed opportunities for improving postpartum family planning coverage. As indicated in the World Health Organization's publication on programming strategies for postpartum family planning (16), an understanding of both the health system in terms of how it is structured, organised, staffed, and financed as well as current government policies is essential to assess existing gaps and opportunities for offering family planning to postpartum women. Using standardised instruments and tools to identify and address each client's needs for family planning services during delivery or child immunisation would be critical. The focus for integration of postpartum family planning services and other health services should be on services that are at the point of childbirth or in the period immediately following. If family planning services are not available at the time of contact with the health system, women should be referred to facilities where they could access the family planning services that could not be provided to them immediately.

### Limitations

The measurement of family planning based on the reproductive calendar likely suffers from large recall errors. In Nigeria, where the modern contraceptive prevalence was already very low, postpartum family planning was even lower, which further limits meaningful disaggregation and analysis.

## Conclusions

This paper highlights the importance of understanding the demographic and social determinants, as well as the sub-national differences in modern postpartum contraception use in Ethiopia, Malawi, and Nigeria. There were effects by age group, but no significant variation by marital status, parity (with the exception of Ethiopia), or birth interval. There are pervasive equity issues in the use of postpartum family planning by education level, place of residence, and wealth quintile, but postpartum family planning behaviours seem more connected to the nexus of service delivery. In this study, opportunistic contacts with the health system through childbirth in facilities and child immunisation were significantly associated with the adoption of postpartum family planning and thus potentially represent the greatest currently missed opportunities for integration.
